# Development of a New Procedure for Evaluating Working Postures: An Application in a Manufacturing Company

**DOI:** 10.3390/ijerph192215423

**Published:** 2022-11-21

**Authors:** Davide Gattamelata, Mario Fargnoli

**Affiliations:** 1 Italian Workers’ Compensation Authority (INAIL), Via Fontana Candida 1, Monte Porzio Catone, 00078 Rome, Italy; 2 Engineering Department, Universitas Mercatorum, Piazza Mattei 10, 00186 Rome, Italy

**Keywords:** ergonomics, occupational health and safety (OHS), risk assessment, task analysis, working postures, ISO 11226 standard, musculoskeletal diseases (MSDs), manufacturing plant

## Abstract

Musculoskeletal diseases represent a constant phenomenon in occupational health and safety (OHS) despite the large effort at governmental and technical levels. In the industrial context, numerous studies have dealt with the evaluation of the physical demand of workers. Moreover, numerous studies have investigated the problem, providing tools for ergonomics analysis. However, practical approaches aimed at integrating ergonomics issues in risk assessment activities are still scarce. To reduce such a gap, the current study proposes a procedure for the evaluation of the static working postures of workers to be included in the risk assessment activities. Such an approach is based on the ISO 11226 standard, providing a practical checklist that can be used both at the workstation’s design stage and during risk assessment activities. Its effectiveness was verified through a case study at a manufacturing company. The results achieved showed that as well as the non-conformity of the workstations’ design, the lack of training of the operators on how to maintain a neutral posture while working can also lead to awkward postures of the trunk and head. Additionally, the proposed methodology allowed us to verify the correctness of each workstation based on the physical characteristics of the workers, providing a useful guideline for the company managers on how to properly assign working tasks.

## 1. Introduction

All over the world in recent years, governments have promoted the achievement of a safer working environment by means of the issue of ever-stricter safety technical standards and regulations following the so-called “zero accident vision” [1]. Such an approach consists of fostering a safety culture through ever-safer behaviors, which can lead to a workplace where no workers are injured or affected by diseases that can cause disability [2,3]. However, despite such an effort, the number of accidents and diseases has not substantially diminished: for example, data reported by Eurostat show that although a decreasing trend can be observed, considering both accidents and diseases in the EU Member States in the period 2013–2019 at a general level [4,5], it should be noted that:

- a considerable number of accidents related to the use of work equipment and machinery can still be observed;
- diseases classified in the musculoskeletal disorders category showed no decrease in the period 2013–2019.

**Figure 1:**
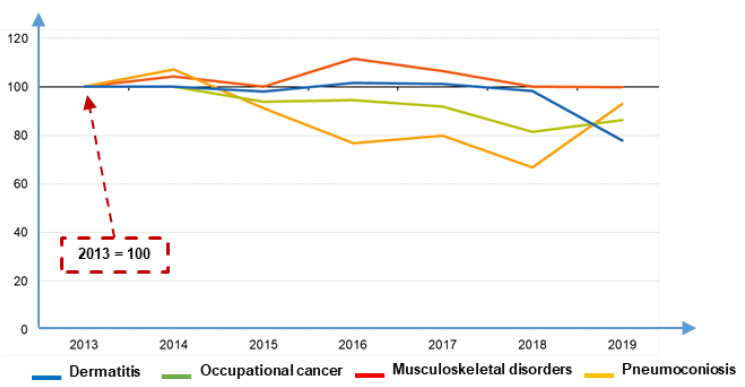
Musculoskeletal diseases statistics (elaborated from [5]).

In Figure 1, the trend of musculoskeletal disorders compared to other occupational diseases in the EU countries is reported, where data are normalized with respect to the number of cases registered in 2013 [5].

The output of these data is in line with recent studies in the field of machinery safety and ergonomics [6], which indicate that most injury-causing events are associated with working tasks’ management and technical safety measures [7,8,9,10]. As stressed by different authors [11,12], when developing a product or work equipment, engineers hardly take into account the final user’s perspective by including the feedback of the users’ experience in design activities. Such a lack has the potential to reduce the product/machinery quality and its safety during usage [13,14]. Moreover, damages due to exposure to awkward work environments negatively impact the workers’ employment, causing a significant social problem [15,16].

To deal with this problem in an effective manner, a thorough approach to risk assessment is deemed necessary, which can put together the design of workplaces and work equipment with the analysis of working tasks and workers’ behavior while operating it [17,18]. In other words, to achieve a safe interaction between the operator and the work equipment he/she is called to use daily, an analysis of the organizational, environmental, and job factors is needed, concentrating on those ergonomic features impacting the operator’s behavior, such as human and individual characteristics [19]. In the extant literature, the need to focus on human factors in industrial engineering has been brought to light in different studies [20,21,22], stressing the need to integrate the analysis of risk factors related to work equipment with that concerning workstation design and human behavior. Such an issue is also fostered by occupational health and safety (OHS) and machinery safety regulations, where the analysis of risks related to both work equipment misuse and the potential variability of the users should be taken into account [23].

Several approaches dealing with the evaluation of the physical demands of workers can be found in the literature, although those addressing the manufacturing sector are limited [24,25]. In such a context, both the Rapid Upper Limb Assessment (RULA) and Rapid Entire Body Assessment (REBA) methods are the most widespread to evaluate the risk of work-related musculoskeletal disorders (WMSDs) [26]. In particular, the RULA method is aimed at providing a rapid evaluation of the loads caused by the postures of the neck, trunk, and upper limbs, while the REBA method was found to be more sensitive to musculoskeletal risks in the evaluation of postures in health care and other service industries [27].

Other approaches are those promoted by public bodies, such as, for example, the Physical Demand Assessment (PDA) tool [28], which consists of a procedure aimed at gathering data related to the physical demands of a certain working task in specific areas of the human body, based on the identification of ergonomic risks in the plant’s production lines [24]. Such a tool provides a framework of a general nature for the qualitative evaluation of ergonomic risks, while its practical implementation requires a case-by-case adaptation to a workstation. Another well-known tool is represented by the so-called “NIOSH equation”, which allows engineers to carry out a task analysis of the manual materials handling exposures that involve lifting and lowering operations [29]. Such a tool has been used worldwide as a reference guide for the evaluation of the recommended weight limit (RWL) for lifting tasks, especially in its revised version that includes the assessment of asymmetry and coupling factors. This is often used in combination with RULA and REBA tools for a complete evaluation of musculoskeletal disorders (MSDs) in workplaces, but since it was developed based on the anthropometric features of the North American population, an adaptation could be required if the anthropometric distributions of the investigated sample are different [30].

However, as well as the diffusion of these tools and the large number of studies dealing with the assessment of working postures, several authors have recognized the need for further research on the development of more practical approaches. In particular, de Galvez et al. [7] emphasized the necessity of providing practical assessment tools aimed at facilitating the integration of ergonomics issues into occupational risk assessment procedures. Similarly, Li et al. [31] outlined that limited research has been conducted on the evaluation of the working condition and physical demand in the manufacturing industry, where one of the most significant ergonomic risk factors is represented by the awkward body posture of operators. Accordingly, they promoted the use of more thorough approaches capable of capturing all information needed for risk assessment (e.g., the task duration). Indeed, an inadequate working posture can lead to musculoskeletal disorders, which are usually associated with pain and fatigue. Such a condition can even influence the posture of workers causing errors that can lead to an increase in hazardous situations and a decrease in the quality of working performances [32].

From the technical point of view, the ISO 11226 standard [33] (which was reviewed and confirmed in 2018) proposes a two-stage procedure for the assessment of workers’ static postures, taking into account five different postures related to the trunk, head, shoulder, forearm/hand, and legs. For each one of these elements, the standard provides technical information on how to carry out the acceptability assessment. However, this information is fragmented and the majority of the suggested criteria might be difficult to understand for non-expert evaluators, reducing its applicability in practical contexts.

The present study aims at contributing to reducing the above-mentioned gaps by proposing a procedure for the evaluation of operators’ static working postures to be included in the risk assessment activities. In more detail, the proposed tool consists of an augmentation of the requirements provided by the ISO 11226 standard and its application has been carried out at a manufacturing company.

## 2. Materials and Methods

Current ergonomic risk assessment tools do not allow engineers to perform objective and quantitative evaluations since they require further information to be implemented practically [26,31]. To overcome this limitation, a procedure for the risk assessment of the static working postures was implemented merging the recommendations of the ISO 11226 standard within the traditional risk assessment approach. As observed by Pinto et al. [34], most occupational risk assessment (ORA) tools are characterized by the following stages:

1. Hazards identification
2. Risks estimation
3. Risks prioritization.

**Figure 2:**
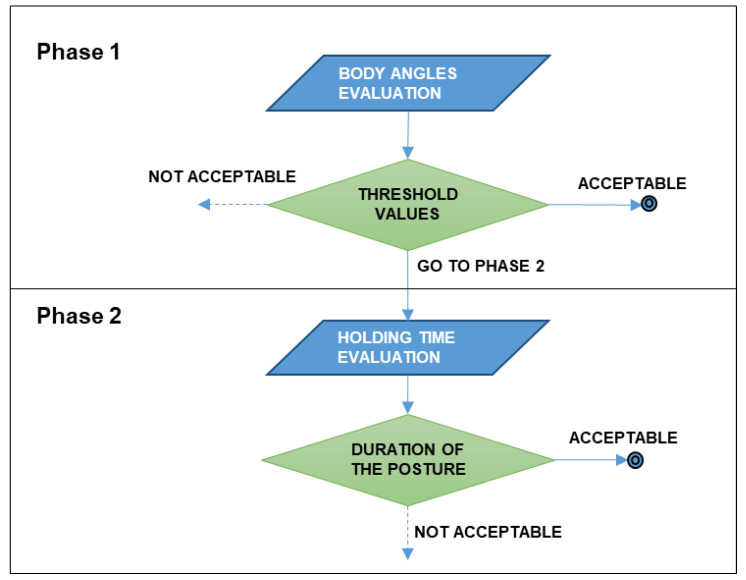
Scheme of the assessment procedure provided by the ISO 11226 standard.

These methods usually rely on data that are subject to imprecision because of the lack of information, as well as the scarce use of expert judgments; all factors that can lead to subjective and incorrect interpretations of results [35]. Hence, the use of the ISO 11226 standard, which is a worldwide accepted technical reference for the assessment of static working postures, can certainly reduce the above-mentioned flaws by providing more reliable results. The ISO 11226 standard proposes a procedure aimed at the evaluation of workers’ static postures to determine whether they can be considered acceptable or not [36]. In such a context it must be noted that a “static working posture” is a “posture maintained longer than 4 s”; another definition that should be borne in mind is that related to the “reference posture”, which can be defined as the working posture where the worker’s upright trunk is non-rotated, his/her arms hang freely, and his/her head allows him/her to look straight forward [33]. The posture can be considered both when the worker is standing and when sitting, while body angles can be classified into three categories: awkward, moderate, and neutral [37]. In more detail, for postures held for more than 4 s, the procedure proposed by the ISO 11226 standard consists of two main phases: in the first phase, the worker’s body angles are evaluated, and the threshold values can be acceptable, not acceptable, or “go to phase 2”. The latter recommendation means that an evaluation of the duration of the static working posture (i.e., the “holding time”) is needed, which represents the second phase of the procedure (Figure 2).

Such a simple procedure should be repeated to evaluate the postures related to the trunk, head, shoulder, forearm/hand, and legs, respectively. However, the applicability of this approach is limited by the quality of the information provided, as outlined among others by Delleman and Dul [38].

To overcome these difficulties in this study, a checklist was developed, where for each evaluation element the assessment criteria are derived from the standard, and practical information on how to perform the measurements is provided.

The checklist is divided into five parts, where the following notations are adopted:

- TR = evaluation of the trunk posture.
- α = angle representing the trunk’s flexion.
- HE = evaluation of the head posture.
- β = angle representing the head’s flexion.
- SH = evaluation of the shoulder posture.
- γ = angle representing the shoulder’s flexion.
- AR = evaluation of the forearm/hand posture.
- δ = angle representing the forearm flexion.
- LE = evaluation of the leg posture.
- ε = angle representing the leg flexion.

The estimation of the above angles should be carried out considering the difference between the rest posture and the working posture. The output of each evaluation consists of the suggestion of one of the actions reported in Table 1.

**Figure 3:**
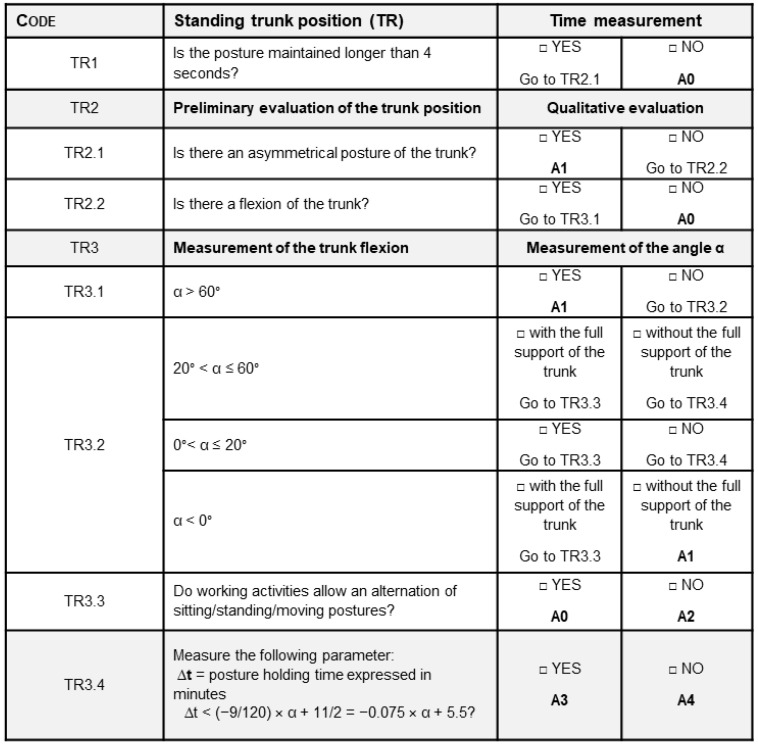
Excerpt of the checklist for the ergonomic risk assessment of the trunk in a standing position, where: A0 stands for “acceptable”; A1 means “not recommended—workstation redesign”; A2 stands for “not recommended—workflow redesign”; A3 means “not recommended—need for recovery time”; A4 means “not recommended—reduction of holding time”.

Figure 3 shows an excerpt of the checklist concerning the evaluation of trunk position while standing, where:

- TR2.1 refers to the asymmetrical position of the trunk caused by the torsion of the backbone and/or the lateral flexion of the thorax against the pelvis.
- TR2.2 refers to the longitudinal flexion with respect to the vertical line.
- TR3.4 refers to the maximum holding time of the posture.

In more detail, the formula provided at stage TR3.4 was elaborated starting from the diagram provided by the ISO 11226 standard, which defines the maximum holding time with respect to trunk flexion. In such a diagram, different areas are individuated depending on the value of the α angle and the holding time, where the latter takes into account both the recovery time between one holding time and the following one, as well as the number of holding-time/recovery-time cycles. These parameters were calculated using the diagrams provided by the ISO 11226 standard considering a working day (8 h). Accordingly, the calculation of the maximum holding time requires engineers to interpolate these parameters to find the correct position in the diagram.

To simplify such a process, in the proposed procedure, we considered the threshold values in the acceptable range of values of the α angle only, translating it into a linear equation. In other words, while the diagram provided by the ISO 11226 standard considers all the possible situations, the formula reported in Figure 3 takes into account only the acceptable values of the posture holding time, making the assessment procedure easier to be used.

**Figure 4:**
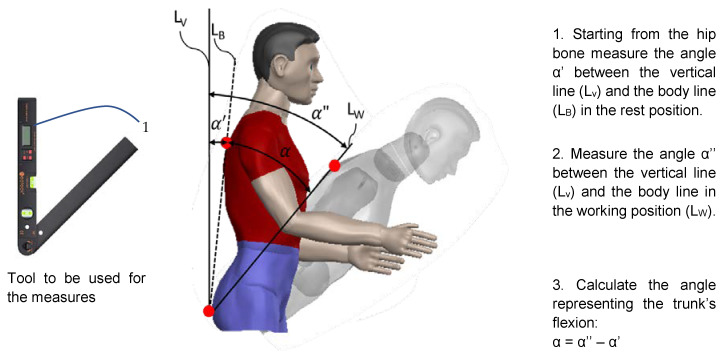
Practical procedure to measure trunk flexion.

Moreover, information on how to practically measure the angle representing the trunk’s flexion (α) is also included in the checklist as shown in Figure 4: in particular, the use of a digital protractor is suggested to measure the angles. This aspect again differs from the ISO 11226 standard, where no specific requirements on how to make the measurements are provided.

**Figure 5:**
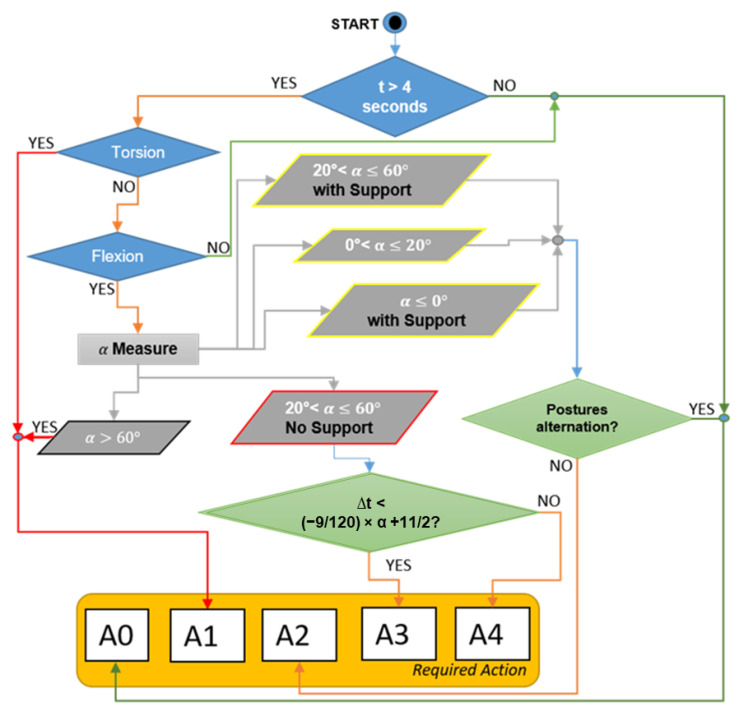
Overview of the procedure to evaluate the posture of the trunk, where: A0 stands for “acceptable”; A1 means “not recommended—workstation redesign”; A2 stands for “not recommended—workflow redesign”; A3 means “not recommended—need for recovery time”; A4 means “not recommended—reduction of holding time”.

The whole procedure for the evaluation of the trunk’s posture is schematized in Figure 5: unlike the ISO 11226 standard, a step-by-step process is provided that leads directly to the definition of a required action.

**Figure 6:**
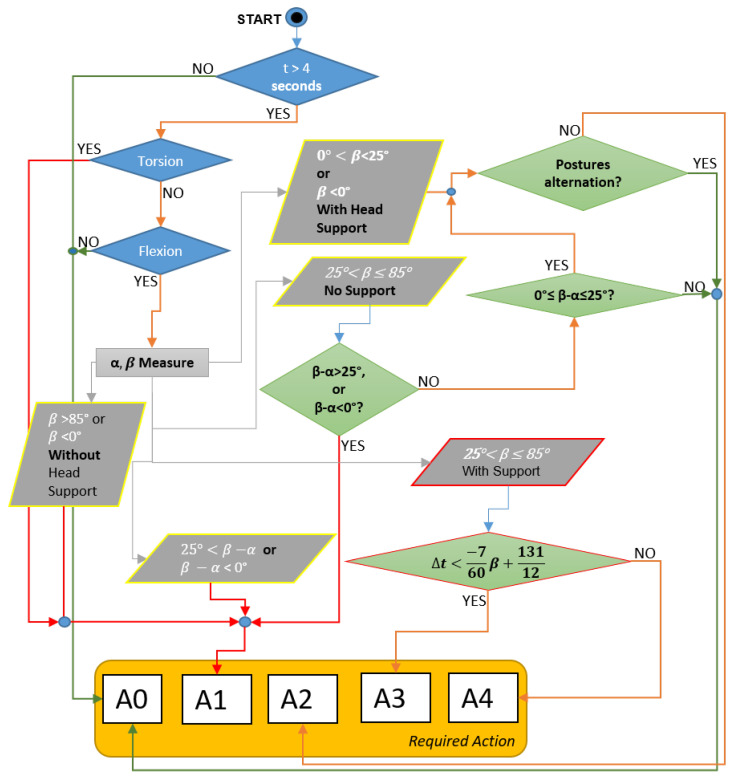
Overview of the procedure to evaluate the posture of the head, where: A0 stands for “acceptable”; A1 means “not recommended—workstation redesign”; A2 stands for “not recommended—workflow redesign”; A3 means “not recommended—need for recovery time”; A4 means “not recommended—reduction of holding time”.

Likewise, the checklist provides instructions on how to evaluate the posture of the head, shoulders, forearm/hand, and legs: as an example, in Figure 6, a scheme of the procedure to evaluate the head posture and the related angle β is shown.

**Figure 7:**
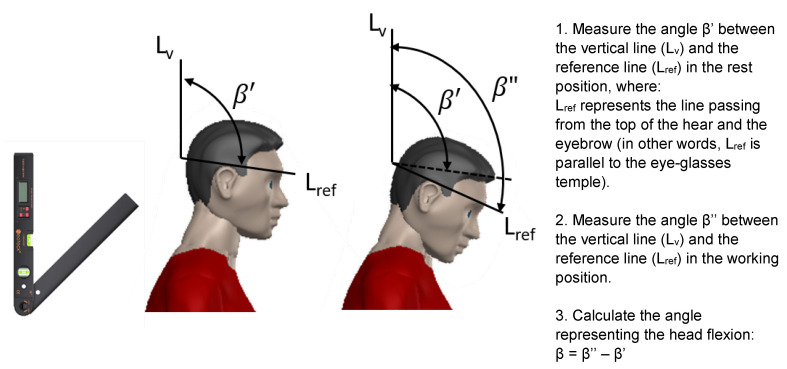
Practical procedure to measure head flexion.

As far as the measurement of the angle β is concerned, the instructions seen in the following figure (Figure 7) were included in the checklist.

When the evaluation is completed, improvement solutions can be adopted, ranging from the redesign of workstations and equipment to specific training activities on how to use the work equipment and further ergonomics analyses (e.g., manual handling).

**Figure 8:**
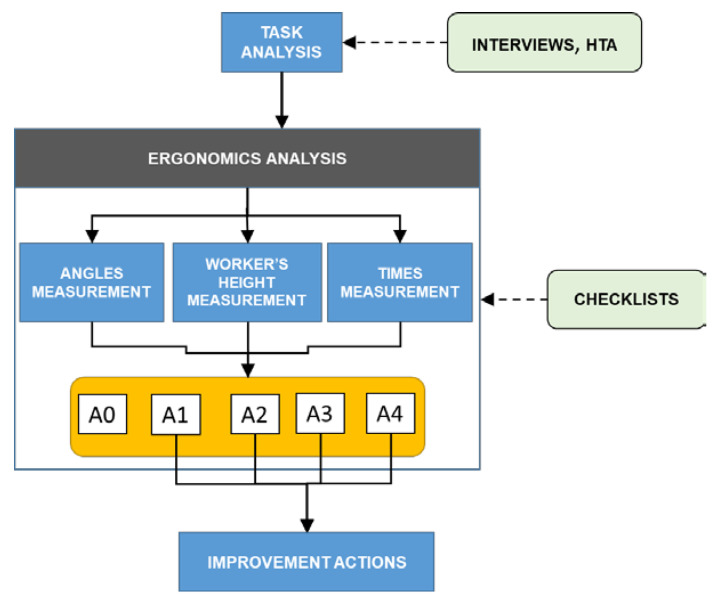
Scheme of the proposed procedure for ergonomic risk assessment of static working postures, where: A0 stands for “acceptable”; A1 means “not recommended—workstation redesign”; A2 stands for “not recommended—workflow redesign”; A3 means “not recommended—need for recovery time”; A4 means “not recommended—reduction of holding time”.

To summarize, the whole process starts with the identification of working tasks and activities: an infield analysis is needed to bring to light the sequence of the tasks at each workstation. For this purpose, interviews with workers and the use of the Hierarchical Task Analysis (HTA) technique [39] can be beneficial. In particular, the “tree-shaped” structure of the latter allows for the breaking down of the working tasks into more detailed subtasks and elementary tasks in a hierarchical manner [40]. Based on this, the static working postures can be identified, and the five checklists are applied by measuring times and angles practically. Thus, depending on the results obtained, improvement actions can be defined, as illustrated in Figure 8, which summarizes the overall flow of the proposed procedure.

## 3. Case Study

The procedure was implemented and verified through a case study at a manufacturing company operating in the automotive sector. The company has different production lines and for each line, workstations were analyzed following the procedure schematized in Figure 8. The analysis considered 11 different workstations, where 50 people work in 3 shifts. Due to a non-disclosure agreement (NDA) with the company, some information provided in this section was simplified.

**Figure 9:**
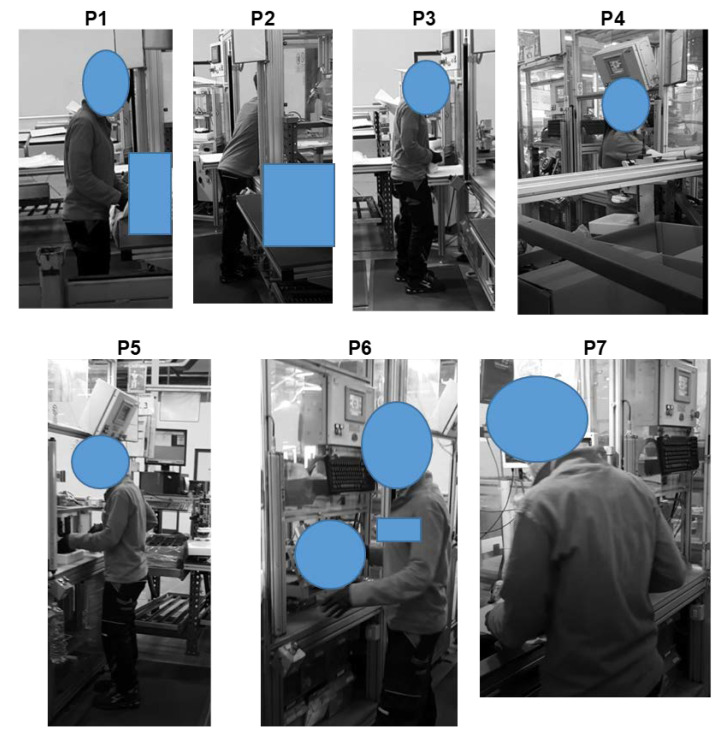
Sequential positions and analyzed postures of the worker in the Alpha workstation.

The details of the Alpha workstation, as illustrated in Figure 9, are characterized by seven different sequential positions of the worker, which correspond to the elementary tasks obtained by the task analysis carried out by means of the HTA method. It must be noted that position P4 is seldom carried out.

**Figure 10:**
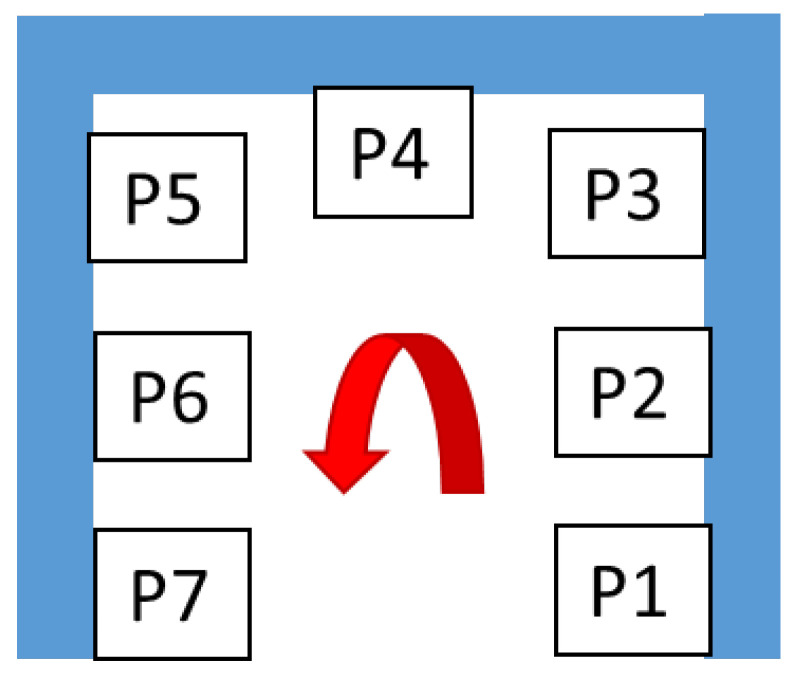
The layout of the positions in the Alpha workstation.

The Alpha workstation is “U” shaped and three different workers use it alternatively (in three different work shifts) to perform manual assembling operations supported by machinery. In Figure 10, the layout of this workstation is schematized.

After the task analysis was completed, the measurement of the body flexions during each elementary task was carried out, also taking into account how long each position is maintained. Hence, the checklist was filled twice to evaluate the postures of each worker. The output of the analysis is reported in Appendix A (Table A1, Table A2, Table A3, Table A4 and Table A5).

**Figure 11:**
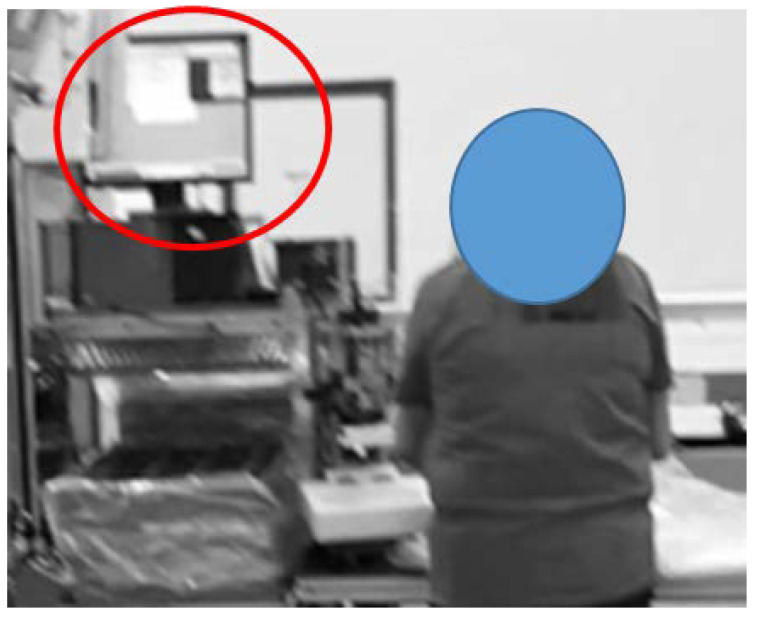
The criticality that emerged in P4 of the Alpha workstation.

The analysis was repeated considering the stature of the other two operators. In particular, repeating the measurements for a worker whose height is less than 165 cm, a criticality emerged in Position 4, where the worker has to flex his/her head excessively to read the data on a monitor (β − α < 0°) leading to an A1 non-conformity (Figure 11). In this case, the redesign of the equipment is required so that the position of the fixed monitor is comfortable for all the workers. Hence, providing the possibility to adjust the distance of the monitor depending on the worker’s stature can be a solution allowing all the workers a neutral position.

**Figure 12:**
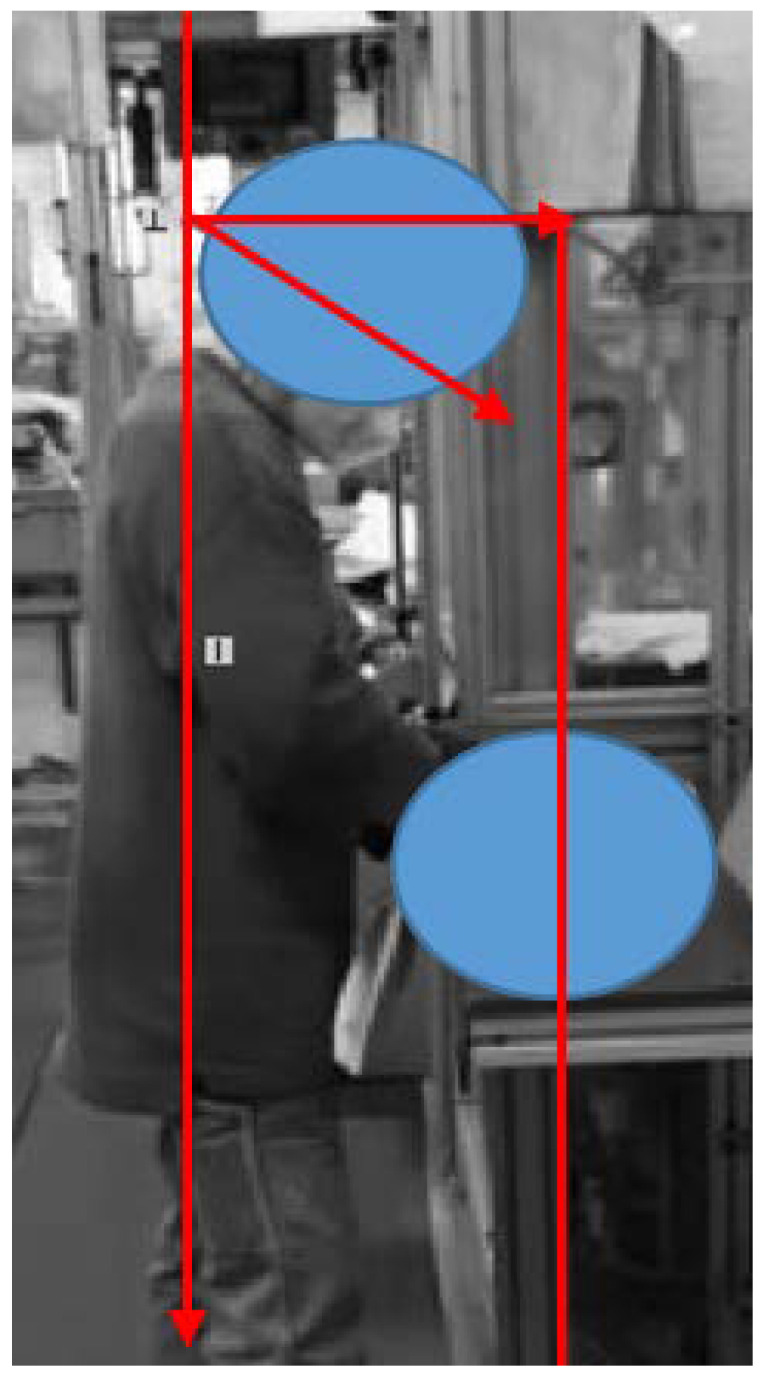
Critical flexion of the head in the Beta workstation.

A similar non-conformity was found in the analysis of the other lines, where the inappropriate height of the workbench led to a critical flexion of the head (β − α > 25°) as shown in Figure 12. In this case, the recommendation is to allow the raising of the workbench. However, this workstation can also be critical for workers whose height is ≥185 cm. Hence, rather than redesigning the whole workstation, a solution can consist of limiting the access to this workstation to workers whose height is compatible with the workbench and equipment dimensions.

**Figure 13:**
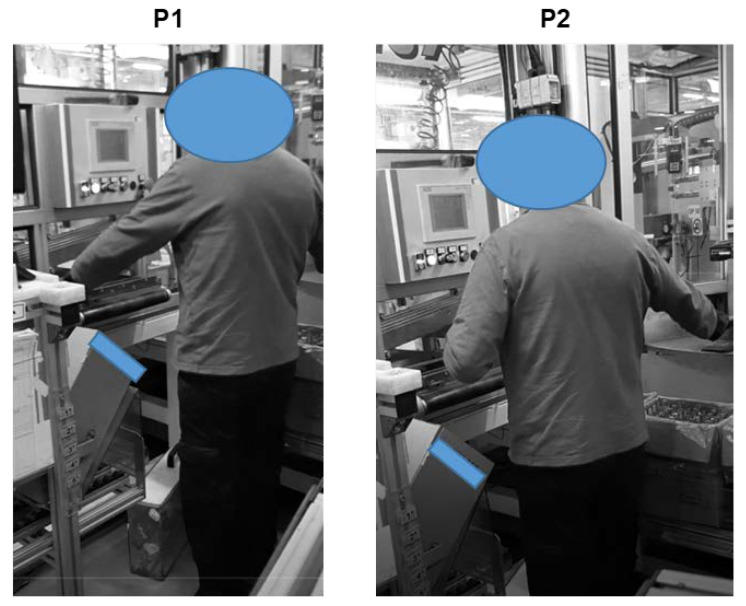
Critical torsion of the trunk in the Gamma workstation.

Another critical situation was found in the packaging area, where a critical torsion of the trunk of the worker emerged (Figure 13). In more detail, in the Gamma workstation, which is provided with two consecutive and orthogonal positions, it was noticed that some workers twist their trunk to move from one position to the next one, while others turn the trunk completely, where the latter corresponds to the correct movement.

In this case, the recommendation consisted of providing the workers with specific training activities in order to achieve a neutral working posture.

## 4. Discussion

**Figure 14:**
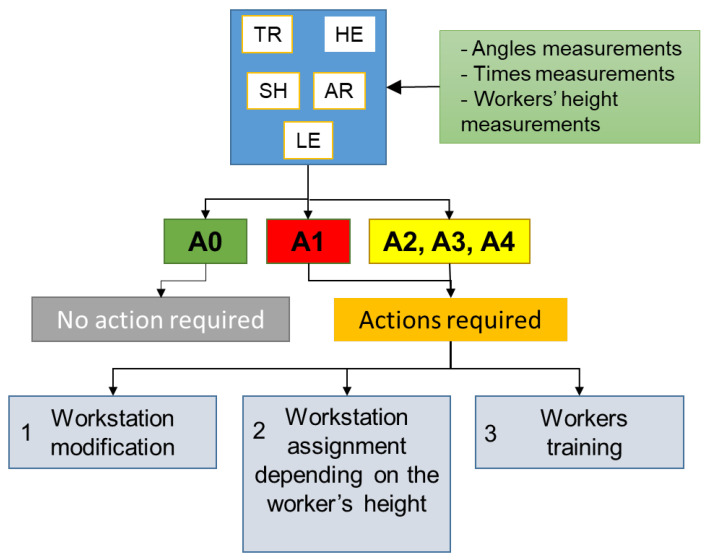
Case study output: detail of the ergonomic assessment parameters and posture risk reduction.

To summarize, 11 workstations were analyzed, and the use of the checklist allowed us to bring to light three major criticalities related to the trunk and head posture. The diagram in Figure 14 outlines the risk assessment, analysis, and reduction process adopted in the current study.

According to the evaluation carried out, three main issues emerged for the reduction of ergonomic risks related to static postures:

1. Workstation modification: this criticality was found in more than one workstation, bringing to light the relevance of design activities, which should take into account not only the generic task that is performed but also the elementary activities that the workers have to carry out to complete it. These results are consistent with previous studies on workers within the automotive manufacturing industry, where the most relevant exposure to MSDs involved both the neck and shoulder [41]. In particular, assembly tasks resulted as the most critical in line with the research findings by Colim et al. [42]. Dantan et al. [43] proposed a design framework that relies on the analysis of the function–behavior–structure to consider human factors in the design of production processes, bringing to light the interactions between workers and equipment. This aspect is relevant to the gaining of information on how awkward positions and fatigue, as well as other factors, affect work activities. Clearly, several studies have proposed tools and methods aimed at modelling equipment and human interaction to reduce exposure to awkward work environments, thus reducing the probability of errors and accidents [44,45] while augmenting productivity [46]. In such a context, the use of digital technologies and models to analyze and redesign workplaces and work equipment has been largely investigated both in the manufacturing industry [47,48] and in other working contexts [49,50,51,52], representing a promising path to achieve human-centred solutions.
2. Workstation assignment depending on the worker’s height: according to occupational health and safety [53] and machinery safety regulations [54], the assignment of a workstation/work equipment has to be carried out considering not only the risks of accidents arising from both their foreseeable use and misuse, but also the physical variability and performances of workers (e.g., stature, physical strength, etc.). While in the analyzed company the number of workers is sufficient to allow a selection on their physical features (e.g., avoiding taller or shorter operators assigned to a certain workstation), this issue can be very critical in other contexts, such as small-sized companies, where the number of workers is very limited and it is not possible to replace workers’ assignments. However, the latter should be considered a stopgap measure and the modification of the workstation/work equipment should always be foreseen, as pointed out by Hernandez-Arellano et al. [55], who suggested the improvement of a workstation based on workers’ anthropometric data as to allow shorter and taller workers its safe use. Moreover, considering that workers of different statures are assigned to the same task and position, the need to include the workers’ height as one of the main parameters for the evaluation of static working postures emerged. This aspect is consistent with studies underlying the importance of considering the variability of operators that are assigned to a specific task/equipment to accommodate worker size and task requirements [56].
3. Workers’ training: during the postural investigation, another criticality emerged concerning the different ways in which each worker performs the same task. Such situations require informative and training actions that can correct the wrong behavior of workers, helping them to always maintain the correct posture. Training the workers to correctly perform manual tasks can be beneficial to make them aware of unsafe postures as pointed out by Soumitry and Teizer [57]. In line with these findings, Colim et al. [58] stressed the importance of augmenting the awareness and training of workers regarding ergonomics and correct posture/handling techniques. Accordingly, it is essential to provide workers with specific training on the proper work techniques and safe working postures. As observed among others by Burgess-Limerick [59], ergonomics training should involve not only beginners but also experienced workers, and their involvement can be improved by specific participatory ergonomics programs.

These issues can be considered as the three main lines of intervention to reduce the workers’ exposure in manufacturing companies, confirming the findings of previous research in this field [60,61].

From the methodological point of view, the proposed procedure allowed us to provide a detailed ergonomics analysis of the static postures, bringing to light specific improvement solutions for each elementary task. Hence, it can be considered an advancement compared to existing tools. Indeed, on the one hand, it relies on the scientific soundness of the ISO 11226 standard; on the other hand, unlike the standard, it provides a simpler step-by-step process that guides engineers to the measurement of postures and indicates for each situation the specific required action. Such a process starts with task analysis, allowing a clearer distinction between subtasks and elementary tasks, considering their proper sequence. Practical information is then provided on how to measure the different angles by means of a digital protractor, and reference values are indicated by means of simple equations to achieve the proper required action. The process of the ergonomic parameters’ estimation can be implemented in a software application to make the results immediately available. Therefore, its use in a knowledge management system for occupational risk assessment is already planned, facilitating the creation of a company’s memory of safety management factors such as work environments and individual characteristics [62,63].

With respect to observational evaluation methods, the proposed procedure includes the practical measurement of each working situation, augmenting the effectiveness of the results in line with research suggestions by Burdorf [64]. This is also consistent with the research hints by Tee [26], who observed that most methods are observational or survey-based tools that assess the risk of ergonomics without proper measurement. In particular, unlike the checklist proposed by Li et al. [31] the measurement of times is included in the procedure allowing for more precise results. The procedure can be applied both at the design stage (in this case, the measurements should consider different population percentiles), and as a reverse engineering approach during periodical risk assessment activities.

To summarize, it can be concluded that the proposed procedure:

1. provides a user-friendly method for the evaluation of static postures at the workplace;
2. integrates times and workers’ variability evaluations, allowing for more specific and precise results;
3. is based on the task analysis, allowing the decomposition of the tasks to reduce exposure;
4. provides specific improvement solutions;
5. provides information that can be used for workers’ training.

As well as these positive aspects, some limitations also need to be considered. Firstly, the procedure has been applied to one case study only. Hence, to augment its validity, further applications are needed in different working contexts. Moreover, to take into account the workers’ variability, different measurements have to be repeated, which can result in a time-consuming process. It must also be stressed that the proposed approach is aimed at evaluating static working postures only, while other tools have to be applied for a more comprehensive analysis of workers’ exposure to MSDs [65,66].

In addition, it must be pointed out that the proposed procedure does not systematically provide direct identification of the causes that determine non-recommended postures. Actually, even though in some work situations (for example, the ones illustrated in the case study) it is possible to easily identify the main causes of non-recommended postures, in other types of work activities, this might not be easy to deduce. Hence, at a general level, a subsequent phase of analysis with specific methods is necessary.

## 5. Conclusions

Work-related MSDs represent a criticality for many industries and ergonomic analysis should always be included in risk assessment activities to reduce the workers’ exposition. In this study, we focused our attention on static working postures, which involve the whole body of the worker and are very common in different types of working contexts. Starting from the analysis of international technical standards, we proposed a procedure that allows managers and engineers to evaluate static working postures in a simplified manner, leading them to precise and specific improvement solutions. Such an approach was verified by means of a case study in a manufacturing company and the findings brought to light that as well as the workstation redesign, the consideration of the variability of workers also plays a fundamental role in reducing exposure. Another relevant finding consists of the importance of training workers in executing work tasks by assuming a correct posture. Indeed, the execution of simple and elementary tasks is often underestimated from the ergonomics point of view, leading workers to assume awkward postures that can have serious consequences on their health.

The results achieved could be useful for the implementation of new or modified MSD risk assessment tools. However, to extend the validity of these first results further, applications involving different working contexts are certainly needed.

## Figures and Tables

**Table 1:** Required actions for risk reduction.

Code	Meaning	Required Action
A0	The posture is acceptable	A further ergonomic assessment of the static posture is not needed
A1	Not recommended	The workstation needs to be redesigned and new information and training activities have to be provided to workers in order to achieve a neutral working posture
A2	Not recommended	The flow of working tasks shall be redesigned by alternating standing/sitting/moving postures
A3	Not recommended	A recovery time shall be foreseen in the flow of work activities.
A4	Not recommended	The holding time should be reduced

## Appendix A

In this appendix the details of the application of the checklist in the workstation alpha are reported, divided as follows:

- Table A1: output of the trunk posture evaluation;
- Table A2: output of the head posture evaluation;
- Table A3: output of the shoulder posture evaluation;
- Table A4: output of the forearm and arm posture evaluation;
- Table A5: output of the legs posture evaluation.

**Table A1:** Trunk posture evaluation.

Worker 1		Worker Height: 170 cm													
Position		P1		P2		P3		P4		P5		P6		P7	
Equipment Height (cm)		92		100		105		103		103		98		97	
TR	Trunk Posture	P1		P2		P3		P4		P5		P6		P7	
TR1	Is the posture maintained longer than 4 s?	YES	Go to TR2.1	YES	Go to TR2.1	YES	Go to TR2.1	NO	A0	NO	A0	NO	A0	NO	A0
TR2.1	Is there an axial torsion of the trunk?	NO	Go to TR2.2	NO	Go to TR2.2	NO	Go to TR2.2	NO	Go to TR2.2	NO	Go to TR2.2	NO	Go to TR2.2	NO	Go to TR2.2
TR2.2	Is there an axial flexion of the trunk?	YES	Go to TR3	YES	Go to TR3	YES	Go to TR3	YES	Go to TR3	YES	Go to TR3	YES	Go to TR3	YES	Go to TR3
TR3	α′ (degrees)	3		3		3									
	α″ (degrees)	5		20		6									
	α″ − α′ = α (degrees)	2		17		7									
TR3.1	α > 60°	NO	Go to TR3.2	NO	Go to TR3.2	NO	Go to TR3.2								
TR3.2	20° < α ≤ 60°	YES	Go to TR3.3	YES	Go to TR3.3	YES	Go to TR3.3								
	0° < α ≤ 20°														
	α < 0°														
TR3.3	Do working activities allow an alternation of sitting/standing/moving postures?	YES	A0	Si	A0	Si	A0								
TR3.4	∆t (min) *	0.17		0.18		0.15									

* In this case, the evaluation foreseen by the step TR4 was not necessary.

**Table A2:** Head posture evaluation.

HE	Head Posture	P1		P2		P3		P4		P5		P6		P7	
HE1	Is the posture maintained longer than 4 s?	YES	Go to HE2	YES	Go to HE2	YES	Go to HE2	NO	A0	NO	A0	NO	A0	NO	A0
HE2	Is there a lateral flexion of the head?	YES	Go to HE3	YES	Go to HE3	YES	Go to HE3								
HE3	Β′ (degrees)	88.30		88.30		88.30									
	Β″ (degrees)	113.90		123.00		119.00									
	β = β″ − β′ (degrees)	25.60		34.70		30.70									
HE4	Is β > 85° or β < 0° without the full support of the trunk?	NO	Go to HE5	NO	Go to HE5	NO	Go to HE5								
HE5	Is 0°< β ≤ 25° or β < 0° with the full support of the trunk?	NO	Go to HE7	NO	Go to HE7	NO	Go to HE7								
HE6	Do working activities allow an alternation of sitting/standing/moving postures?	YES	A0	YES	A0	YES	A0								
HE7	Is 25°< β ≤ 85° without the full support of the trunk?	YES	Go to HE9	YES	Go to HE9	YES	Go to HE9								
HE8	Is 25°< β ≤ 85° with the full support of the trunk?	-	-	-	-	-	-	-	-	-	-	-	-		
HE9	One of the following conditions occurs: β − α > 25°, or β − α < 0°	NO	Go to HE10	NO	Go to HE10	NO	Go to HE10								
HE10	One of the following conditions occurs: 0° < β − α < 25°?	YES	Go to HE6	YES	Go to HE6	YES	Go to HE6								

**Table A3:** Shoulder posture evaluation.

SH	Shoulder Posture	P1		P2		P3		P4		P5		P6		P7		
SH1	Is the posture maintained longer than 4 s?	NO	A0	NO	A0	NO	A0	NO	A0	NO	A0	NO	A0	NO	A0	
SH2	Does the elbow assume an inappropriate position?	NO	A0	NO	A0	NO	A0	NO	A0	NO	A0	NO	A0	NO	A0	

**Table A4:** Forearm and arm posture evaluation.

AR	Forearm and Arm Posture	P1		P2		P3		P4		P5		P6		P7		
AR1	Is the posture maintained longer than 4 s?	NO	A0	NO	A0	NO	A0	NO	A0	NO	A0	NO	A0	NO	A0	

**Table A5:** Legs posture evaluation.

LE	Legs Posture	P1		P2		P3		P4		P5		P6		P7	
LE1	Is the posture maintained longer than 4 s?	YES	Go to LE2	YES	Go to LE2	YES	Go to LE2	YES	Go to LE2	YES	Go to LE2	YES	Go to LE2	YES	Go to LE2
LE2	Is verified as one of the following situations: -an extreme flexion of the knee. -a plantar flexion > 20° or dorsiflexion of the ankle > 50°. -while standing there is a flexion of the knee < 180°.	NO	A0	NO	A0	NO	A0	NO	A0	NO	A0	to	A0	to	A0
